# Assessment of flavor characteristics in snakehead (*Ophiocephalus argus* Cantor) surimi gels affected by atmospheric cold plasma treatment using GC-IMS

**DOI:** 10.3389/fnut.2022.1086426

**Published:** 2023-01-11

**Authors:** Jia-bao Huang, Xian-wang Kong, Ying-yun Chen, Jing Chen

**Affiliations:** ^1^College of Food and Pharmacy, Zhejiang Ocean University, Zhoushan, China; ^2^Key Laboratory of Health Risk Factors for Seafood of Zhejiang Province, Zhoushan, China; ^3^School of Petrochemical Engineering & Environment, Zhejiang Ocean University, Zhoushan, China

**Keywords:** atmospheric cold plasma, snakehead, surimi gels, gas chromatography-ion mobility spectrometry (GC-IMS), flavor characteristics

## Abstract

The gel formation ability of freshwater surimi is weak, resulting in its poor flavor and quality. Atmospheric cold plasma (ACP), a widely developed non-thermal processing technology in the food industry, is considered to have potential applications in maintaining and improving the flavor characteristics of surimi gels. In this study, the effect of ACP on snakehead surimi gels flavor at different treatment times was investigated by sensory evaluation and gas chromatography-ion mobility spectrometry (GC-IMS) analysis. The results showed that ACP could better maintain and improve the original appearance and tissue state characteristics of surimi gels, scoring about 1–2 points higher than the ACP-untreated group. GC-IMS analysis demonstrated the obvious difference in the volatile organic compounds (VOCs) among the treatment groups. Specifically, the samples treated for 120 s with ACP exhibited the most unique aroma characteristics, which probably related to the highest thiobarbituric acid reactive substances values (73.28 μmol MDA/kg sample). Meanwhile, the reduced TCA-soluble peptides content indicated that ACP could inhibit protein degradation to maintaining the tissue state and flavor characteristics of the surimi gels. In conclusion, the advantages of ACP treatment, such as little damage to nutrients, and maximum retention of original sensory properties, provide new ideas for its application in the flavor characteristics of the snakehead surimi gels.

## 1. Introduction

Concentrated myofibrillar protein of surimi is the primary material for making surimi gels (1). Surimi gel products, such as fish balls, fish tofu, and crab sticks, are loved for their high nutrition, high protein, elastic texture, and unique flavor characteristics. As seawater fish resources have dwindled, freshwater fish has become a potential raw material for surimi gel products (2). Snakehead (*Ophiocephalus argus* Cantor), an being extensively farmed and processed freshwater fish, is a good candidate for surimi based products due to its excellent nutrients and high-quality protein. However, snakehead is underutilized. Snakehead processed into surimi can boost its economic value and provide a new raw material for the surimi industry. Nevertheless, compared to seawater surimi, freshwater surimi contains unpleasant aroma compounds, coupled with high levels of myoglobin, which makes consumers uncomfortable (3). Therefore, adopting appropriate methods to solve the problems of poor aroma and low whiteness is vital to obtaining certified freshwater surimi gels.

Traditional approach to remove surimi smell is rinsing. This method is economical and effective, and the different rinsing methods have a significant effect on odor elimination, affecting the scent distribution of surimi gels (4). Nevertheless, rinsing will destroy the substances in the fish to a certain extent, and has unavoidable disadvantages in the study of the aroma properties of surimi gels. So it is impossible to obtain the specific and accurate information, which hinders the high-quality processing of surimi gels. Therefore, it is urgent to combine a surimi processing method with odor detection technologies to tackle these challenges.

Atmospheric cold plasma (ACP) is a non-thermal technology that uses ionized gases to preserve food and maintain mass without destroying its original composition. Ionized gases constitute highly excited ions and reactive species, such as gas atoms, free radicals, and quanta of ultraviolet (UV) radiation (5). Plasma contains a large number of charged particles, reactive oxygen species (ROS), reactive nitrogen species (RNS), excited molecules, UV photons, positive and negative ions (6). ROS includes superoxide anion, atomic oxygen, singlet oxygen, ozone, hydroxyl radical, and RNS contains nitrogen, atomic nitrogen, nitric nitrogen, UV photons, positive and negative ions. ROS oxidation is one of the most important outcomes of protein exposure to plasma. Oxidation can cause protein fragmentation, cross-linking, unfolding, and conformation changes (7). The thiol groups cysteine and methionine in the hydrolyzed products are oxidized by ROS, which maintains the protein’s tertiary structure, improving gelation and quality. ROS attacks on lipids can lead to the formation of secondary oxidation products such as aldehydes, which produce odors and affect the sensory quality. In general, lipid oxidation tends to be more pronounced with higher plasma voltage and longer treatment times (8), resulting in a more obvious effect on the flavor. Flavor studies of different aquatic products using ACP have been widely reported by many researchers. Pérez-Andrés et al. (9) found that ACP could promote the carbonyl formation of mackerel protein and accelerate protein oxidation, which changed the flavor. Olatunde et al. (10) confirmed that plasma could induce oxidation of a myofibrillar protein isolate of Asian sea bass, which is manifested as a slight reduction of sulfhydryl content, an increase of carbonyl content, and a change of surface hydrophobicity, thus changing the gel properties and sensory properties of the protein. Additionally, the results showed that ACP could maintain sensory quality in chub mackerel by slowing down bacterial proliferation and reducing the production of volatile bases and oxidized compounds (11). However, little information is available on the specific odorant composition of snakehead surimi gels after ACP treatment, so further investigations should be considered.

Volatile organic compounds (VOCs) are important components in food and have a great influence on the aroma of food. For precise fragrance information, instrumental analysis is more suitable for the detection of VOCs than sensory analysis at the molecular level (12). GC-ion mobility spectrometry (GC-IMS) is a widely utilized and effective food aroma analysis technology that can accurately quantitatively and qualitatively analyze VOCs in surimi gels. The two-dimensional separation model of GC-IMS has good responsiveness and high separation accuracy, and has a high sensitivity to compounds with strong proton affinity, resulting in a low detection limit and high detection sensitivity for alcohols, carboxylic acids, and nitroalkanes (13). Recent research on food aromas has combined this approach (14), which detected 62 volatile compounds in sturgeon by GC-IMS, mainly alcohols and esters. In addition, Goggin et al. (15) confirmed that the combination of GC-IMS can reveal the aroma information of food more reliably, comprehensively, and scientifically. However, as far as we know, few studies have established the association assessment of surimi gels aroma characteristics with ACP for improved gel performance based on GC-IMS analysis.

This study aimed to analyze the aroma characteristics in snakehead surimi gels as affected by different ACP treatment times (60, 90, and 120 s) using GC-IMS analysis and to specifically explore the changes of color and aroma properties of surimi gels by sensory evaluation and principal component analysis (PCA). This study would have a practical significance for providing useful knowledge for improving the quality of snakehead and other freshwater surimi and their products and providing a reference for the food industry to use ACP technology for treatment.

## 2. Materials and methods

### 2.1. Materials and reagents

Fresh snakeheads (*O. argus* Cantor, 1.5–2 kg) were kindly obtained from the Laoqi market (Zhoushan, China), while edible salt and food-grade polyethylene enteric casings were purchased from the Hello City Supermarket (Zhoushan, China). All reagents were of analytical reagent grade and provided by Sinopharm Group Co., Ltd. (Shanghai, China).

### 2.2. Preparation of surimi gels

All fish were transported to the laboratory within 30 min in a plastic water tank specially equipped with enriching oxygen and adequate ice. The head, guts, bones, scales, and skin were removed, and the white meat of the snakehead was collected. The white meat was then rinsed to remove surface impurities. According to Zhou et al. (16), the white muscle was chopped for 3 min into surimi with a blender (Supor, Shaoxing, Zhejiang, China), then 2.5% (w/w) edible salt was added to the surimi paste and chopped for another 1 min. The surimi paste was filled into a polyethylene sausage casing (20 mm in diameter) before being sealed on both ends tightly, and heated at 40° for 60 min and 90° for 30 min. The heated surimi sausages were cooled in ice water and then stored in the refrigerator at 4° overnight until further determination.

### 2.3. Atmospheric cold plasma treatment

**FIGURE 1:**
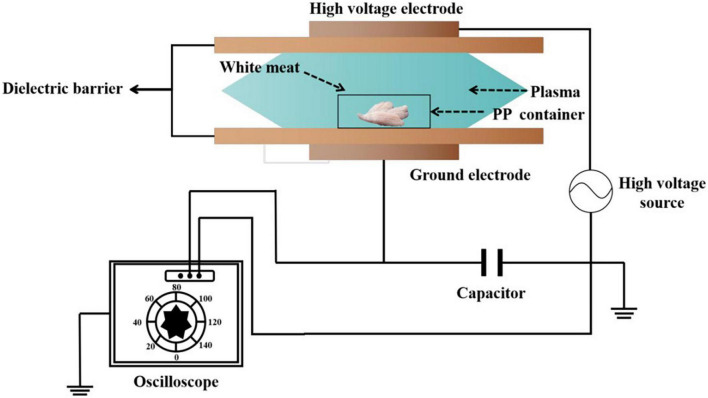
Schematic diagram of dielectric barrier discharge operation.

Atmospheric cold plasma (ACP) with a dielectric barrier discharge (DBD) plasma system (BK130/36, Phenix Technologies, Campbell, USA) was used to treat the surimi samples (Figure 1) (17). The DBD device was comprised of two parallel rounded aluminum plate electrodes, and the distance between both electrodes was 38 mm. A dielectric layer is placed between the two electrodes. A high voltage transformer with an output voltage of 0–50 kV was used to deliver the necessary energy required for the generation of reactive species (RS) from atmospheric gases. White meat, approximately 0.5 cm thick, was placed between electrodes in a 15-cm length, 8-cm width, and 2-cm height polypropylene container. The treatment temperature was close to room temperature (25–30°C). The samples were subjected to a voltage of 40 kV for various treatment times (60, 90, and 120 s). Control samples without ACP treatment have been considered. All samples were stored on ice until they were utilized within 2 h to avoid unnecessary spoilage.

### 2.4. Sensory evaluation

Ten panelists (5 men and 5 women) were selected based on their availability, expertise in the food industry, and prior experience on trained panels. The appearance, tissue state, taste, and smell of surimi gel samples were evaluated according to the sensory evaluation criteria listed in Supplementary Table 1, with the proportions of 0.2, 0.2, 0.3, and 0.3, respectively.

### 2.5. Determination of whiteness

Whiteness includes L* (lightness), a* (redness/greenness), and b* (yellowness/blueness) values of the surimi gels, which were measured using a colorimeter (CR-400, Konica Minolta, Tokyo, Japan). Whiteness was calculated based on the following equation:


$$\text{Whiteness}=100-{\left[{\left(100-L^{*}\right)}^{2}+a^{*2}+b^{*2}\right]}^{1/2}$$

### 2.6. Determination of electronic nose (E-nose)

Electronic nose measurement was performed using a portable E-nose analysis system (PEN 3, Germany). According to the method of Xu et al. (18), the surimi gel was cut into small cubes (2 g) and then packed into a 50 ml centrifuge tube and sealed with two layers of clingfilm immediately. The detection time lasted for 120 s, and the E-nose system was cleaned once before each sample test. Each measurement was repeated three times under the same conditions.

### 2.7. Determination of gas chromatography-ion mobility spectrometry (GC-IMS)

In this research, a GC-IMS device (FlavourSpec^®^, G.A.S., Dortmund, Germany), and an Agilent 490 gas chromatograph (Agilent Technologies Inc., CA, USA) were used to examine the dynamic change of volatile components in surimi gels. Each sample was replicated three times.

The surimi gels (2 g per sample) were transferred to a 20 ml headspace vial and then incubated at 60°C for 20 min before automatic sampling. After that, 500 μl headspace was injected into a capillary column (WAX, 30 m × 0.53 mm ID, G.A.S., Dortmund, Germany) at a syringe temperature of 60°C using nitrogen as a carrier gas under the following programmed flow: the flow rate was 2 ml/min initially, 2 ml/min at 2 min, 10 ml/min at 10 min, 100 ml/min at 20 min, and finally up to 150 ml/min at 25 min before termination. Drift gas (nitrogen) was set at a constant flow rate of 150 ml/min. The Reporter and Gallery Plot plug-ins were used to make the different signal peaks and fingerprint maps of volatile organic compounds in surimi gels. The NIST and IMS databases in the instrument software were performed to do a qualitative analysis of volatile compounds (14).

### 2.8. Determination of thiobarbituric acid reactive substances (TBARS)

The surimi gel (5 g) was homogenized with 25 ml of trichloroacetic acid (TCA) solution (7.5%, v/v), then filtered through two filter papers with the same volume of thiobarbituric acid solution (TBA) (0.02 mol/L), and heated at 90°C for 30 min (19). After cooling with ice water for 15 min, the wavelength was measured at 532 nm. TBA values were rendered by the change in malonaldehyde (MDA) content in surimi gels.

### 2.9. Determination of TCA (trichloroacetic acid)-soluble peptides

TCA-soluble peptides reflect small molecule peptides in fish surimi gels and hence the degree of protein hydrolysis under endogenous cathepsin, whose content is negatively associated with protein degradation (20). Referring to the method of Chaijan et al. (20), 5 g of surimi gels were mixed with 45 ml of 5% (w/v) TCA, and the mixture was homogenized for 1 min. The homogenates were maintained at 4°C for 60 min and then centrifuged for 10 min at 5,000 rpm. The peptide content in the supernatant was determined according to the BCA protein assay kit (P0011, Shanghai, China) method.

### 2.10. Statistical analysis

All the experimental data were analyzed by IBM SPSS 22.0 statistical software (SPSS Inc., Chicago, USA) and expressed as mean ± standard deviation. Graphs were constructed using Origin 2021b (Origin Lab Inc., MA, USA). The determination of significant differences (*p* < 0.05) between groups was based on Analysis of variance (ANOVA) and Duncan’s multiple range.

## 3. Results and discussion

### 3.1. Sensory evaluation analysis

**FIGURE 2:**
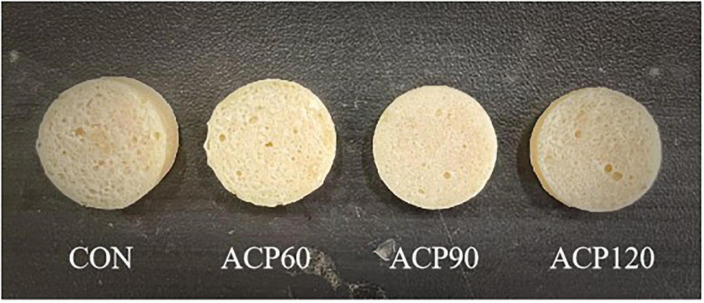
Appearance of surimi gels at different treatment times with ACP. (CON, surimi gels without ACP treatment; ACP60, surimi gels treated with ACP for 60 s; ACP90, surimi gels treated with ACP for 90 s; ACP120, surimi gels treated with ACP for 120 s).

**FIGURE 3:**
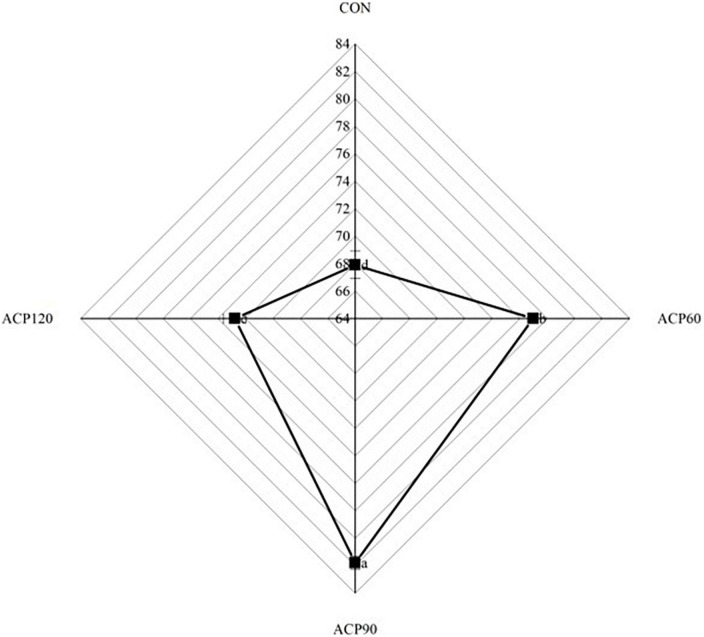
Whiteness of surimi gels without and with ACP treatment. The different lowercase letters represent significant differences (*p* < 0.05). (CON, surimi gels without ACP treatment; ACP60, surimi gels treated with ACP for 60 s; ACP90, surimi gels treated with ACP for 90 s; ACP120, surimi gels treated with ACP for 120 s).

The appearance characteristic is one of the essential factors affecting the sensory perception and consumer food acceptance of the surimi gel flavor (21). Sensory evaluations of snakehead surimi gels, untreated and treated by ACP at various times, are shown in Table 1. The sensory score was the highest for the CON (6.88) sample group, followed by the ACP60 (6.85), ACP90 (6.69), and ACP120 (6.52) sample groups. In Figure 2 and Table 2, it can be noticed that the ACP60, ACP90, and ACP120 sample groups had a greater score than the CON sample group in appearance and texture. This was coincidental with the better whiteness in ACP-treated sample groups (Figure 3). This might be due to the formation of ROS such as hydroxyl radicals on the surface of surimi gels and the oxidative modification reaction of pigment compounds in fish, and these active substances changed the color of surimi gels’ appearance (22). At the same time, the presence of ozone and reactive oxygen species free radicals could destroy the porphyrin structure of heme pigments (mainly myoglobin and hemoglobin) in fish (23). Moreover, ozone could improve the sensory properties of the gels by making the samples whiter, brighter, and more transparent than before. Similar results were observed for ozone-treated silver carp surimi (24). However, the CON sample group expressed a higher score than the other sample groups in taste and odor. The main reason was that the reactive species degraded some of the organic components of the gels, thus creating a strong ozone and grass smell (25). This is congruent with the findings of Chen et al. (11), who discovered that mackerel odor scores reduced as the number of days following ACP therapy increased and that scores of around 6.00 after 12 days were generally acceptable. Therefore, the sensory scores of samples after ACP treatment in this study were still acceptable. Sensory judgment issues created by group members were also a possibility.

**TABLE 1:** E-nose sensors and its corresponding representative sensitive compounds.

Sensors	Sensitive compounds
W1C	Aromatic
W5S	Nitrogen oxide compounds
W3C	Ammonia, aromatic ingredients
W6S	Hydride
W5C	Olefes, aromatic molecules
W1S	Methane
W1W	Sulfide
W2S	Ethanol, partial aromatic compounds
W2W	Aromatic ingredients, organic sulfide
W3S	Alkane, aliphatic series

**TABLE 2:** Sensory evaluation of surimi gels without and with atmospheric cold plasma (ACP) treatment.

Sample	Appearance	Tissue state	Taste	Smell	Score
CON	5.50 ± 1.05^d^	6.10 ± 1.02^d^	7.24 ± 0.85^a^	7.95 ± 1.00^a^	6.88 ± 0.72^a^
ACP60	7.10 ± 1.00^b^	6.90 ± 0.97^c^	7.00 ± 0.35^b^	6.50 ± 1.07^b^	6.85 ± 0.64^a^
ACP90	7.60 ± 0.95^a^	7.34 ± 0.74^a^	6.28 ± 0.80^d^	6.05 ± 0.32^c^	6.69 ± 0.52^b^
ACP120	6.70 ± 0.91^c^	7.10 ± 1.25^b^	6.55 ± 0.98^c^	5.97 ± 0.94^c^	6.52 ± 0.91^c^

*Different superscript letters in the same column indicate significant differences (*p* < 0.05). (CON, surimi gels without ACP treatment; ACP60, surimi gels treated with ACP for 60 s; ACP90, surimi gels treated with ACP for 90 s; ACP120, surimi gels treated with ACP for 120 s).

### 3.2. Electronic nose (E-nose) cluster analysis

**FIGURE 4:**
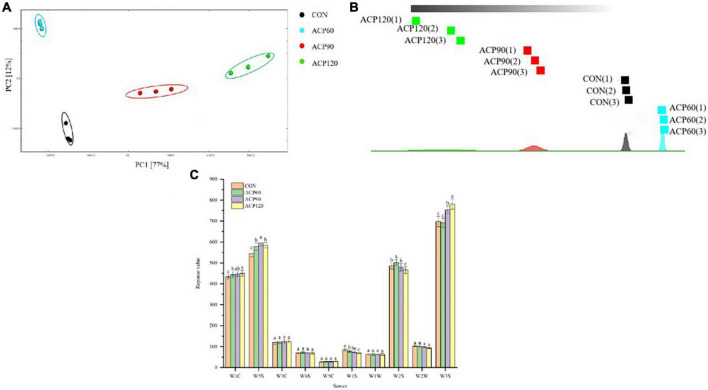
The analysis of PCA **(A)**, nearest neighbor algorithm distance map **(B)**, and response value **(C)** by E-nose of surimi gels subjected to different treatment times. The different lowercase letters represent significant differences (*p* < 0.05). (CON, surimi gels without ACP treatment; ACP60, surimi gels treated with ACP for 60 s; ACP90, surimi gels treated with ACP for 90 s; ACP120, surimi gels treated with ACP for 120 s).

By simulating the human nose’s odor systems, the e-nose measures basic smell sensations to evaluate overall scent requirements objectively. Principle component analysis (PCA) is a statistical method for clustering and analyzing differences between obtained sample data (26). Figure 4A shows the PCA results of the surimi gels after ACP treatment; PC1 (77%) and PC2 (12%) contributed close to 90%, indicating that the two principal components could describe the overall fragrance characteristics of each gel sample. In Figure 4A, different color regions depict the overall flavor distribution of the samples under varied treatment settings. The three points in each region represent three repeated tests for each sample, with the distance between colored regions representing sample group differences. The flavor response distribution areas of the surimi gels were clearly different and did not overlap. This means that the ACP treatment changed the smell and taste of the surimi gels in a significant way (27). Furthermore, combined Figures 4A, B demonstrate that ACP120 samples were farthest from CON samples, followed by ACP90 and ACP60, suggesting that odor differences were positively connected with ACP treatment duration.

The response values of samples without and with ACP treatment are presented in Figure 4C following detection using ten sensors. The sensitive compounds corresponding to the ten sensors are listed in Table 1. The response of the gas-sensitive sensors is affected by the composition concentration, and pressure of the volatile substances, which may vary over time during E-nose sampling. The odor compounds of the snakehead surimi gels were abundant and diverse, as shown in Figure 4C. It was found that significant differences in the response intensity of W1C, W5S, W2S, and W3S (*p* < 0.05) were observed between the four groups of samples, and showed the strongest response to each volatile compound. This result indicated that snakehead surimi gels might have high concentrations of nitrogen oxides, ethanol, and alkanes while being insensitive to ammonia, hydrides, olefins, methane, and sulfide. It was noteworthy that these components in the samples after ACP treatment were significantly different from the CON samples. This is consistent with the findings of Li et al. (28), who reported that the original odor of the plasma-treated samples was obviously distinct from the untreated meatballs.

### 3.3. Gas chromatography-ion mobility spectrometry (GC-IMS) analysis

#### 3.3.1. GC-IMS differential graph analysis

**FIGURE 5:**
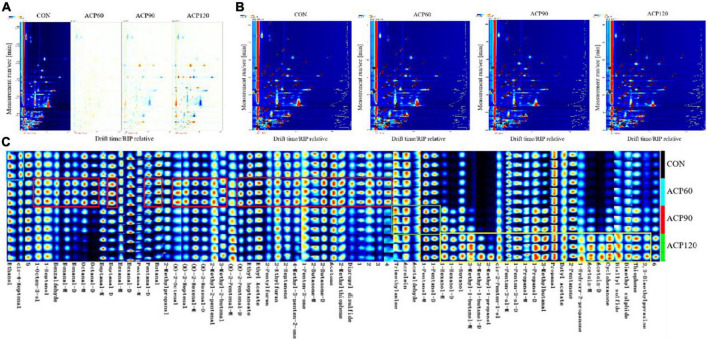
The analysis of two-dimensional differential map **(A)**, qualitative map **(B)** and fingerprint map **(C)** of surimi gels subjected to different treatment times. (CON, surimi gels without ACP treatment; ACP60, surimi gels treated with ACP for 60 s; ACP90, surimi gels treated with ACP for 90 s; ACP120, surimi gels treated with ACP for 120 s).

The GC-IMS method is utilized to obtain overall IMS information from various snakehead surimi gel samples in order to further compare the differences in volatile odor compounds (VOCs) and variation regularities. Generally, the abscissa indicates the ion drift time, the ordinate represents the retention time of the gas chromatography, and each point to the right of the reaction ion peak (RIP) expresses the VOCs. The top views of the two-dimensional different map are shown in Figure 5A after normalizing the ion drift time and the RIP position. If the VOCs are identical, the topographic background deduced from the other samples is represented in white using CON as the reference. In the plot, red dots indicate higher VOCs content than CON, and blue dots mean lower VOCs content than CON. Compared with CON, ACP90 and ACP120 showed an obvious decrease in the content of some VOCs in surimi gels and no significant change in ACP60. The VOCs in the gel might react with some plasma to form new components during ACP treatment, resulting in a reduction in the content of the original VOCs. For ACP60 samples, the short ACP treatment time might not be sufficient to significantly alter the VOCs content. Meanwhile, many red spots were observed in the ACP120 samples, indicating that the VOCs increased or new VOCs formed after ACP treatment. The increase of promoting VOCs might be attributed to protein denaturation, polymerization, and oxidation, as well as lipid oxidation after plasma treatment (29). During the ACP treatment, the texture and moisture of the surimi gels changed, and the network structure of the gels became more stable, thus reducing the water mobility in the gels and leading to an increase in the flavor content. It can be concluded that ACP treatment is able to retain the original VOCs in the surimi gels and can effectively promote the generation of new VOCs. However, the prolonged ACP treatment process could also cause the loss of some VOCs in surimi gels.

### 3.3.2. GC-IMS qualitative analysis

Figure 5B shows the locations of volatile odorants on the qualitative map. The amount of VOCs identified in the gel samples is represented by each point and its corresponding number. GC-IMS was able to find 65 volatile compounds, including 26 aldehydes, 16 alcohols, 10 ketones, 3 esters, 2 furans, 2 thiophenes, and 1 pyrazine, in different samples. These compounds include monomers and partial dimers.

An aroma fingerprint map of the different samples is indicated in Figure 5C. This is a more visible representation of the complete VOCs information for each sample and the difference between samples than the qualitative map. Each column in the figure represents the signal peaks of the same volatile compounds in different samples, while unknown peaks are expressed in numbers. Compounds with distinct differences in concentrations across the groups were framed. Each sample was repeated in triplicate. The analysis showed that the amounts of ethanol and cis-4-heptenal were relatively higher in the samples without ACP treatment. With ACP treatment and the increase of treatment time, the contents of aldehydes (benzaldenyde, E-2-octenal, nonanal, E-2-heptanal, octanal, E-2-hexenal, heptanal, 3-methyl-2-butenal, 2-methyl-2-pentenal, E-2-Pentenal, hexanal, pentanal, 3-methylbutanal, butanal, propanal, acrolein, 2-methylpropanal, acetaldehyde), alcohols (1-octen-3-ol, 1-heptanol, 1-hexanol, 1-pentanol, 1-penten-3-ol, 1-butanol, 1-propanol, 3-methyl-1-butanol, cis-2-penten-1-ol, 2-methyl-1-propanol) and ketones (1-hydroxy-2-propanone, 2-heptanone, 4-methyl-3-penten-2-one, 1-penten-3-one, 2-pentanone, 2-butanone, acetone, cyclohexanone, acetoin) were significantly increased. It is evident that ACP had the greatest impact on aldehyde content. This result was similar to that of Ke et al. (30), who found that aldehydes were the category most affected by the plasma when investigating the effect of cold plasma on the oxidized flavor products of lipids in fish muscles. From the preliminary evaluation in Figure 5C, the 29 compounds shown in the red frame had the highest concentration in the ACP60 group, including 14 aldehydes, 5 ketones, and 2 alcohols. There are 23 compounds as shown in the yellow frame, including 7 alcohols, 3 ketones, 2 aldehydes, 2 ethers, thiophene, and pyrazines, whose contents are not significantly different for the CON group, ACP60 group, and ACP90 group, but clearly higher for the ACP120 group. Four compounds, including trimethylamine, acrolein, acetaldehyde, and 1-pentanol, had similar concentrations in the CON, ACP60, and ACP120 groups, while the concentration was higher in the ACP90 group. Overall, except for ethanol and cis-4-heptenenal, other identified VOCs in ACP-treated gels were present at roughly higher concentrations than CON. Several of these compounds appeared only after ACP treatment, such as 1-hexanol (D), 3-methyl-1-butanol (M and D), 2-methyl-1-propanal, and acetoin (D).

### 3.3.3. GC-IMS quantitative analysis

**FIGURE 6:**
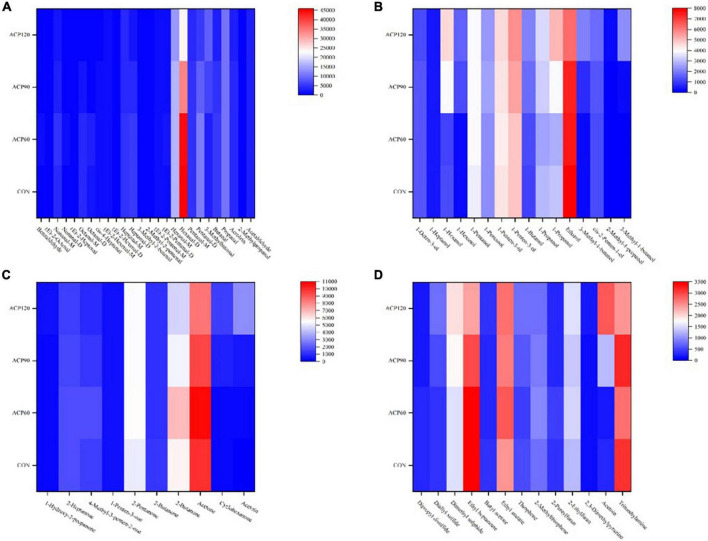
The analysis of volatile organic compounds include aldehydes **(A)**, alcohol **(B)**, ketone **(C)** and Other classes **(D)** by heat map of surimi gels subjected to different treatment times. (CON, surimi gels without ACP treatment; ACP60, surimi gels treated with ACP for 60 s; ACP90, surimi gels treated with ACP for 90 s; ACP120, surimi gels treated with ACP for 120 s).

As shown in Table 3, the formula, retention index, retention time, and drift time of the 65 volatile compounds (including M and D) were all detected. To further quantify the data in Table 3 and the signal intensities of the respective VOCs. The 65 identified compounds were divided into four groups (aldehydes, alcohols, ketones, and others), and their signal intensities heat maps were presented in Figure 6.

**TABLE 3:** Comparisons of the identified volatile compounds in surimi gels by gaschromatography-ion mobility spectrometry (GC-IMS).

Count	Volatile compounds	Formula	RI	Rt [sec]	Dt [RIPrel]	Comment
1	Benzaldehyde	C7H6O	1,547.6	1,409.416	1.15915	
2	1-Octen-3-ol	C8H16O	1,482.4	1,223.339	1.16681	
3	1-Heptanol	C7H16O	1,487.2	1,236.172	1.40981	
4	(E)-2-Octenal	C8H14O	1,436.0	1,106.238	1.33518	
5	Dipropyl disulfide	C6H14S2	1,415.5	1,058.115	1.2663	
6	Nonanal	C9H18O	1,401.3	1,026.032	1.47486	M
7	Nonanal	C9H18O	1,401.3	1,026.032	1.94746	D
8	1-Hexanol	C6H14O	1,367.1	952.551	1.33052	M
9	1-Hexanol	C6H14O	1,369.2	956.872	1.63555	D
10	Ethyl heptanoate	C9H18O2	1,338.5	895.302	1.41868	
11	(E)-2-Heptenal	C7H12O	1,329.5	878.02	1.25647	
12	Octanal	C8H16O	1,296.7	817.53	1.40105	M
13	Octanal	C8H16O	1,296.0	816.45	1.82773	D
14	1-Pentanol	C5H12O	1,264.4	764.601	1.25118	M
15	1-Pentanol	C5H12O	1,263.7	763.521	1.51036	D
16	cis-4-Heptenal	C7H12O	1,254.1	748.399	1.14539	
17	2-Pentylfuran	C9H14O	1,240.6	727.876	1.25294	
18	(E)-2-Hexenal	C6H10O	1,229.8	711.673	1.18065	M
19	(E)-2-Hexenal	C6H10O	1,232.0	714.913	1.51918	D
20	1-Hydroxy-2-propanone	C3H6O2	1,313.4	847.775	1.06076	
21	Acetoin	C4H8O2	1,297.3	818.61	1.05723	M
22	Heptanal	C7H14O	1,194.0	660.905	1.33405	D
23	Heptanal	C7H14O	1,195.6	663.065	1.69197	D
24	2-Heptanone	C7H14O	1,188.3	650.103	1.26176	
25	1-Penten-3-ol	C5H10O	1,175.0	622.019	0.94263	M
26	1-Penten-3-ol	C5H10O	1,175.0	622.019	1.35168	D
27	3-Methyl-2-butenal	C5H8O	1,212.7	686.949	1.09477	
28	2-Methyl-2-pentenal	C6H10O	1,166.4	604.432	1.158	
29	1-Butanol	C4H10O	1,159.7	591.153	1.18006	
30	(E)-2-Pentenal	C5H8O	1,148.8	570.287	1.10653	M
31	(E)-2-Pentenal	C5H8O	1,148.3	569.339	1.36239	D
32	Hexanal	C6H12O	1,096.3	479.234	1.26093	M
33	Hexanal	C6H12O	1,097.0	480.183	1.5609	D
34	4-Methyl-3-penten-2-one	C6H10O	1,113.2	506.74	1.45503	
35	Diallyl sulfide	C6H10S	1,143.8	560.802	1.11683	
36	Butyl acetate	C6H12O2	1,083.4	461.213	1.25211	
37	1-Propanol	C3H8O	1,051.4	419.481	1.11094	M
38	1-Propanol	C3H8O	1,052.2	420.429	1.24769	D
39	Thiophene	C4H4S	1,027.7	391.027	1.03448	
40	1-Penten-3-one	C5H8O	1,039.0	404.305	1.0786	
41	Pentanal	C5H10O	996.9	356.882	1.18888	M
42	Pentanal	C5H10O	997.8	357.83	1.42562	D
43	2-Pentanone	C5H10O	997.8	357.83	1.35798	
44	2-Ethylfuran	C6H8O	965.4	332.222	1.04183	
45	Ethanol	C2H6O	940.7	314.201	1.12712	
46	3-Methylbutanal	C5H10O	927.1	304.716	1.40209	
47	2-Butanone	C4H8O	907.3	291.438	1.05801	M
48	2-Butanone	C4H8O	910.2	293.335	1.24475	D
49	Ethyl acetate	C4H8O2	889.6	280.056	1.34033	
50	Butanal	C4H8O	886.6	278.159	1.27857	
51	Trimethylamine	C3H9N	858.5	261.087	1.14771	
52	Acetone	C3H6O	837.0	248.757	1.11389	
53	Propanal	C3H6O	810.9	234.53	1.14182	
54	Acrolein	C3H4O	861.7	262.984	1.05654	
55	2-Methylpropanal	C4H8O	810.9	234.53	1.3021	
56	2-Methylthiophene	C5H6S	1,061.9	432.759	1.03889	
57	Dimethyl sulphide	C2H6S	788.8	223.148	0.95949	
58	Acetaldehyde	C2H4O	761.5	209.87	0.97861	
59	Cyclohexanone	C6H10O	1,296.8	817.837	1.16241	
60	Acetoin	C4H8O2	1,297.9	819.734	1.33592	D
61	3-Methyl-1-butanol	C5H12O	1,220.0	697.382	1.24769	M
62	cis-2-Penten-1-ol	C5H10O	1,336.2	890.869	0.94479	
63	2-Methyl-1-propanol	C4H10O	1,107.0	496.307	1.16682	
64	3-Methyl-1-butanol	C5H12O	1,220.0	697.382	1.49326	D
65	2,3-Dimethylpyrazine	C6H8N2	1,348.3	914.581	1.108	

*M and D indicate monomers and dimers, respectively.

Aldehydes are the main volatile compounds in flavor development, which generally have fresh, fruity, and fatty aromas (31). Among the 26 aldehydes identified, the intensity of hexanal (M and D) was highest in the four sample groups, followed by propanal, and other aldehydes did not differ significantly (Figure 6A). It was worth mentioning that the CON group had the strongest hexanal signal intensity, which gradually weakened after ACP treatment. ACP processing group was reduced instead, which indicated that some acetaldehyde was oxidized to hexanal (32). While ACP60 did not decrease significantly because the ACP treatment time was too short to reach the oxidation phase. A large proportion of these aldehydes were typical lipid oxidation products, such as 3-methylbutanal, 2-methyl-2-pentenal, 3-methyl-2-butenal, E-2-pentenal, E-2-hexenal, and so on (33). With the ACP treatment, the signal strength of these aldehydes had also increased in varying degrees. For example, the intensity of 3-methylbutanal was significantly increased from 3,113 in the CON group to 9,122 in the ACP120 group (*p* < 0.05), and no significant difference (*p* > 0.05) was found between the CON (101) and ACP120 (133) groups of 3-methyl-3-butenal. Similarly, the signal intensity of E-2-pentenal (M) was also significantly higher in the ACP120 group (1,441) than in the CON group (989) (*p* < 0.05). However, there was no significant difference in the intensity of E-2-hexenal (M and D) and 2-methyl-2-pentenal between the ACP120 and CON groups (*p* > 0.05), both only increasing by about 100 in intensity. Overall, all these results suggested that surimi gel samples treated with ACP could promote lipid oxidation and the formation of the above aldehydes.

Alcohols are mainly generated by the thermal oxidation of lipids and are also important products of lipid oxidation (34). The main alcohols in the gels are 1-pentanol, 1-penten-3-ol, 1-propanol, and ethanol, of which 1-penten-3-ol has fruit and vegetable aromas and greatly contributes to the aroma characteristics of snakehead surimi gels (Figure 6B). The high intensity of ethanol means that the smell of the gels is less fishy. In this work, nine other alcohols were also detected: 1-octen-3-ol, 1-heptanol, 1-hexanol (M and D), 1-butanol, 3-methyl-1-butanol (M and D), cis-2-penten-1-ol, and 2-methyl-1-propanol. The intensity of the other volatile compounds was increased or decreased, except that the 1-heptanol concentration was barely unchanged in the four groups (*p* > 0.05). According to published studies, 1-hexanol and 1-propanol were common lipid oxidation products in surimi, reflecting the degree of rancidity in fish. Protein isolates of hybrid catfish surimi showed higher 1-hexanol and 1-propanol concentrations, as well as TBARS values, as found by Phetsang et al. (35). This is consistent with the result of 1-hexanol in surimi gels after ACP treatment in Figure 5C. Similarly, Yu et al. (36) also found this result in the flavor study of emulsified surimi gels. In a word, consistent with aldehydes, ACP-treated surimi gel samples undergo lipid oxidation, which promotes the formation of alcohols.

Ketones are generated by an interaction between the Maillard reaction and lipid degradation. As shown in Figure 6C, ten ketones were identified in this study, of which 2-heptanone is a common lipid oxidation product in surimi gels (37). Similar to some aldehydes and ketones described above, the intensity of this ketone was also the highest in the ACP60 group, followed by the CON group, and the lowest in the ACP90 and ACP120 groups. Among them, only three ketones have aroma characteristics: 2-heptanone (fruit fragrance similar to pear fragrance), 1-penten-3-one (onion fragrance), and 2-pentanone (floral aroma and fruit fragrance). Although the other ketones in this study did not have aroma characteristics, their intensity also generally increased as the duration of ACP exposure increased. Therefore, ACP treatment significantly changed the content of ketones.

In addition to the aldehydes, alcohols, and ketones, 3 ethers, 3 esters, 2 thiophene, 2 furans, 1 pyrazine, and 2 other substances were also detected in our samples. The concentrations of these 13 compounds varied differently after ACP exposure, with 6 compounds being reduced and 7 being increased. Pyrazine intensity increased with increasing ACP treatment, which was associated with the formation of pyrazine precursors in the ACP-treated samples. The compound 2,3-dimethylpyrazine was identified as having the “roasted nuts” scent and was shown to synthesize novel hydrogen-bonded organic solids constructed with various aromatic acids (38). Esters are the main source of sweet and fruity tastes in foods. It has high content and a unique fragrance. Therefore, they have a substantial impact on the flavor properties of the fish products. Ethyl acetate had the lowest intensity in the CON group when compared to the other esters. The reason for this phenomenon may be due to the ethyl acetate alcoholysis induced by the plasma generated from ACP, which produces methyl acetate and ethanol. This corresponded to the high intensity of ethanol in Figure 6B.

In summary, all the results of the GC-IMS indicated that ACP treatment could promote the formation of VOCs and lipid oxidation in snakehead surimi gels, especially for these aldehydes, alcohols, and ketones. Surimi gels treated without ACP also showed some lipid oxidation properties as compared to the CON and ACP-treated samples. This was probably due to all the free radical species that form the plasma bulk and could promote lipid oxidation (39).

### 3.4. TBARS analysis

**FIGURE 7:**
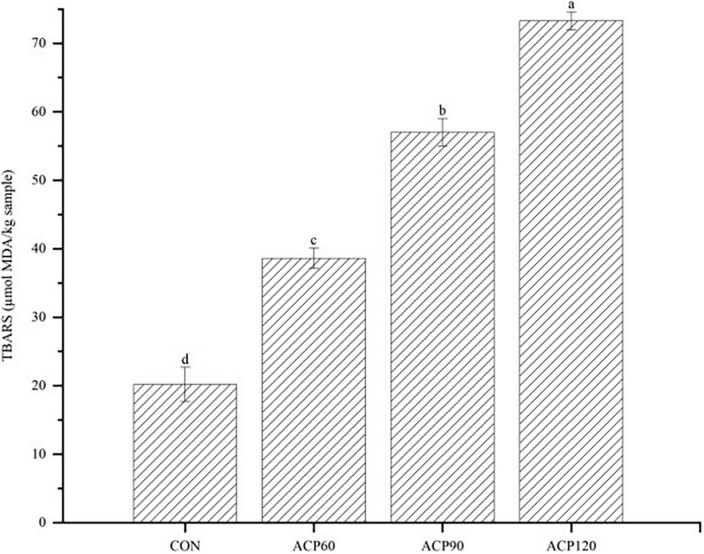
The analysis of TBARS content of surimi gels subjected to different treatment times. The different lowercase letters represent significant differences (*p* < 0.05.) (CON, surimi gels without ACP treatment; ACP60, surimi gels treated with ACP for 60 s; ACP90, surimi gels treated with ACP for 90 s; ACP120, surimi gels treated with ACP for 120 s).

MDA is a derivative of the oxidation and decomposition of unsaturated fatty acids in fish oils, which is one of the indicators to measure the degree of lipid oxidation. The TBARS value can be accurately evaluated for the degree of lipid oxidation by the MDA content. The effects of ACP exposure on the content of TBARS in snakehead surimi gels were presented in Figure 7. It is evident from the figure that the ACP treatment significantly improved the lipid oxidation levels of the samples, which is in line with previous research. For example, Albertos et al. (40) investigated a significant increase in TBARS values from Atlantic herring treated at 80 kV for 5 min. A similar result was found after cold plasma treatment using a corona discharge plasma jet (CDPJ) on Gwamegi (semi-dried raw Pacific saury) at 20 kV (41). In the present study, the TBARS levels were increased from 20.18 μmol MDA/kg sample to 74.28 μmol MDA/kg sample in the CON samples after 120 s of ACP treatment. All samples treated with ACP showed increased TBARS values, a result corresponding to the above GC-IMS VOCs difference map results. It could be found a higher concentration of VOCs in ACP120 group samples. The results of TBARS clearly demonstrated that ACP treatment caused more severe lipid oxidation compared to gels without ACP treatment. Therefore, plasma could cause gels to produce some odor-characteristic organic components that promoted lipid oxidation. The ACP treatment time was increased from 60 to 120 s, with a significant increase in TBARS values (*p* < 0.05), implying that these organic components were mainly generated after 120 s of ACP treatment.

### 3.5. TCA-soluble peptides analysis

**FIGURE 8:**
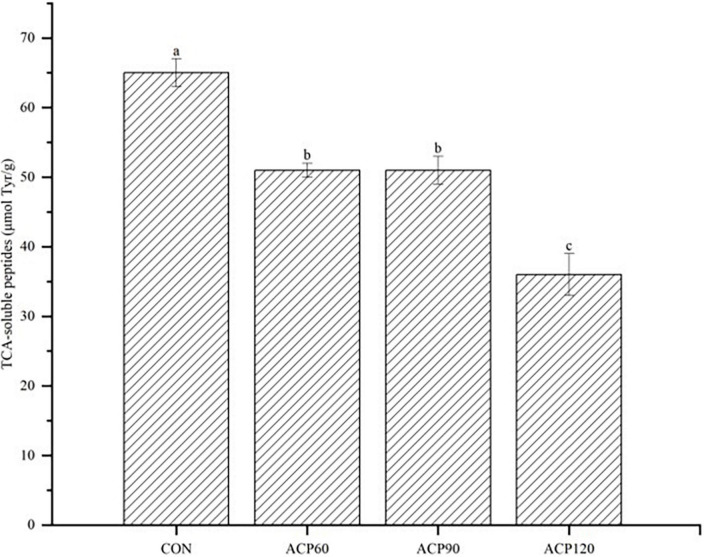
The analysis of TCA-soluble peptides content of surimi gels subjected to different treatment times. The different lowercase letters represent significant differences (*p* < 0.05). (CON, surimi gels without ACP treatment; ACP60, surimi gels treated with ACP for 60 s; ACP90, surimi gels treated with ACP for 90 s; ACP120, surimi gels treated with ACP for 120 s).

Peptides are obtained by proteolysis, and the content of soluble peptides in the hydrolysis products can reflect the efficiency of endogenous proteases’ degradation, thus affecting the flavor characteristics of surimi. TCA-soluble peptides are considered important taste substances. Some peptides have a unique flavor, and peptides have higher digestibility and better flavor than single amino acids (42). Figure 8 shows the TCA-soluble peptides of the surimi gels. ACP treatment significantly reduced the TCA-soluble peptide content in surimi gels (*p* < 0.05), and the value after 120 s of treatment was approximately half of the CON. These results suggested that ACP treatment had a potential inhibitory effect on the release of TCA-soluble peptides. Inactivation of endogenous proteases might cause the gel network structure more stable. Specifically, reactive oxygen species might oxidize free amino acids and proteins, resulting in the chlorination of aromatic groups and hydroxylation of aliphatic amino acid side chains, the nitrification of aromatic amino acid residues, and the conversion of certain amino acid residues (43). Besides, reactive nitrogen species generally affect phenylalanine, tyrosine, cysteine, and methionine, leading to nitrification and oxidation. This oxidation also causes polypeptide chain breaks to form cross-linked protein aggregates (44). This might explain the minimal TCA-soluble peptides obtained in the ACP120 sample. Thus, ACP treatment was able to significantly increase the elasticity of the surimi gels and thus improve the tissue state. However, the taste of the ACP-treated surimi gels slightly decreased. The results were consistent with the conclusions about tissue state and taste in Table 1. The further aggregation of proteins might result in the denaturation of soluble peptides.

## 4. Conclusion

In this study, ACP treatment obviously stimulated mostly shown in the appearance and tissue state. In addition, GC-IMS analysis demonstrated that a total of 65 volatile organic compounds constituted each group of flavors, mainly including aldehydes, alcohols, and ketones. The highest TBARS value would endows the most unique aroma characteristics of the ACP120 group. Moreover, ACP treatment significantly reduced TCA-soluble peptide content. Especially, ACP120 group exhibited the lowest value, which improved the tissue state of the surimi gels. In conclusion, ACP exposure can alter the odor and taste characteristics of surmi gels. It can significantly promote the formation of characteristic volatile flavor substances in surimi gels. This work provides clues for improving the flavor characteristics of snakehead surimi gels induced by ACP.

## Data availability statement

The original contributions presented in this study are included in the article/Supplementary material, further inquiries can be directed to the corresponding author.

## Author contributions

J-BH: conceptualization, data curation, writing – original draft, and writing – review and editing. X-WK: data curation and writing – review and editing. Y-YC: writing – review and editing. JC: funding acquisition, project administration, and validation. All authors contributed to the article and approved the final manuscript.
