# Supplementary material for: Assessment of flavor characteristics in snakehead (Ophiocephalus argus Cantor) surimi gels affected by atmospheric cold plasma treatment using GC-IMS
Source: Front Nutr. 2023 Jan 11;9:1086426. doi: 10.3389/fnut.2022.1086426 (PMC9875017; doi:10.3389/fnut.2022.1086426)
Supplement: Supplementary file 1 [file Table_1.docx]

Supplementary Material

# Table S1. Sensory scoring criteria

| Project | Standard for assessment | Score |  |
| --- | --- | --- | --- |
| Appearance | The surface is very flat and smooth.  The surface is flat with a few small pits.  The surface is more rough, with more small pits.  The surface is more rough, with more large pits.  The surface is very rough, with many pits and large ones. | 9-10  7-8  5-6  3-4  1-2 |  |
| Tissue state | The section is dense, without large pores, with many small and uniform small pores; the middle finger force pressure, obvious depression without rupture, let go can be restored.  The section is dense, without large pores and a few small pores; the middle finger force pressure, depressed and not broken, can be restored.  The section is basically dense, without large pores, with a few small pores; the middle finger force pressure, depression without rupture, and let go can not be completely restored to the original state.  The section is soft with a few uneven holes; the middle finger is cracked  The section is slurry, soft and dense; the middle finger is cracked. | 9-10  7-8  5-6  3-4  1-2 |  |
| Taste | It has an obvious fish flavor.  It has a fish flavor.  No fish fresh flavor, slightly bitter and sour taste.  No fish fresh taste, with a bitter and sour taste.  It is obviously bitter and sour. | 9-10  7-8  5-6  3-4  1-2 |  |
| Smell | It has rich fish aroma.  It has fish aroma.  No fish aroma, with a slight peculiar smell.  No fish aroma, with obvious peculiar smell.  No fish aroma, has a strong peculiar smell. | 9-10  7-8  5-6  3-4  1-2 |  |
